# Circulatory titin and miR-451a are possible sarcopenia biomarkers in elderly people

**DOI:** 10.3389/fragi.2025.1587438

**Published:** 2025-06-12

**Authors:** Roberta Mancuso, Lorenzo Agostino Citterio, Simone Agostini, Rossella Miglioli, Riccardo Nuzzi, Laura Antolini, Fabio Trecate, Mario Clerici

**Affiliations:** ^1^ IRCCS Fondazione Don Carlo Gnocchi ONLUS, Milan, Italy; ^2^ Department of Health Sciences, University of Milano-Bicocca, Milan, Italy; ^3^ Department of Pathophysiology and Transplantation, University of Milan, Milan, Italy

**Keywords:** sarcopenia, biomarkers, titin, microRNA, rehabilitation

## Abstract

**Introduction:**

Sarcopenia is a clinical syndrome characterized by decline of muscle mass, strength or physical performance that occur with advancing age. Diagnosis is currently based on assessment of muscle mass and performance. New biomarkers are needed in clinical practice for diagnosis, monitoring and treatment of sarcopenia. The measurement in urine of titin (TTN), a muscular protein essential for structure and function of sarcomere, has been recently suggested as useful biomarker for the diagnosis of sarcopenia. The titin N-terminal fragment (N-TTN), produced by proteolysis during muscle damage, is released in the bloodstream and is secreted in the urine, and it was suggested as indicator of muscle injury. The primary aim of our study is to evaluate the potential of serum TTN and N-TTN expression as biomarker of sarcopenia, an aspect that has not been the subject of much research so far. Additionally, the secondary aim is to explore possible relationship between the serum expression of titin and miR-451a, its possible miRNA regulator.

**Methods:**

We verified serum TTN, N-TTN and miR-451a concentration in a cohort of 70 sarcopenic patients who were undergoing rehabilitation; results were compared to those obtained in 90 age- and sex-matched healthy controls (HC).

**Results:**

Results showed that TTN and N-terminal TTN (N-TTN) (p < 0.0005 for both) and miR-451a (p < 0.0001) were significantly upregulated in serum of patients compared to HC. Rehabilitation significantly reduced TTN and N-TTN expression (p < 0.05 for all), while induced a significant increase in miR-451a expression (p = 0.008); ROC analysis showed that the change of miR-451a may be a predictive biomarker for rehabilitation outcome (p = 0.0198).

**Discussion:**

This study suggests the involvement of TTN, N-TTN and miR-451a in sarcopenia; moreover, the monitoring of miR-451a concentration may be useful proxy to measure the effectiveness of rehabilitation intervention.

## 1 Introduction

Sarcopenia is an age-associated condition characterized by a gradual loss of skeletal muscle mass, along with reduced muscle strength and functional performance (Cruz-Jentoft et al., 2019).

In the elderly population this disorder represents one of the main health issues, increasing the risk of disability and injures from falls, as well as being responsible for hospitalization, loss of independence and mortality (Cruz-Jentoft and Sayer, 2019). People affected by sarcopenia show several alterations of the neuromuscular junctions (NMJs), featuring a predominance of type I (slow twitch) fibers associated with a decreased size and number of type II (fast twitch) fibers (Kwan, 2013). The shift from fast to slow muscle fibers has significant implications for associated metabolism (aerobic oxidative for type I or instead anaerobic glycolytic for type II) and leads to reduced fatigue resistance and overall muscle performance.

Sarcopenia is also characterized by intermuscular infiltrations of adipose tissue (Laviano et al., 2014), increased production of inflammatory cytokines (Zembron-Lacny et al., 2019), and a decline of muscle satellite cells (MuSC), leading to a reduced muscle regenerative capacity (Sousa-Victor and Munoz-Canoves, 2016).

In the elderly community, sarcopenia can affect 10%–16% of individuals, and its prevalence increases in those over 80 years of age (Yuan and Larsson, 2023); among community-dwelling older adults the prevalence ranges from 10% to 40% (Mayhew et al., 2019). Importantly, the onset of this condition can also occur in middle-aged people: while the disease is primarily associated with ageing, several other factors can contribute to its development; these include physical inactivity, poor nutrition, smoking, extreme sleep duration, and various comorbidities such as diabetes, cancer and neurological disorders (Dhillon and Hasni, 2017). Despite its clinical relevance, the pathophysiology of sarcopenia is only barely clarified, and uniform diagnostic criteria as well as approved pharmacological treatments for this condition are missing. Resistance training, also in combination with adequate protein intake with nutrition (Giacosa et al., 2024), is a crucial component in the rehabilitation of sarcopenia, as it induces several positive clinical effects, such as increasing muscle mass and strength, slowing down the degeneration of skeletal muscles and helping in the regulation of blood sugar and blood pressure (Talar et al., 2021; Giallauria et al., 2016).

Specific biomarkers for sarcopenia are currently lacking, and their development could facilitate the early detection of muscle loss and functional impairment, allowing timely preventive or pharmacological strategies, which could significantly improve the quality of life of these patients.

Potential biomarkers of sarcopenia include the giant sarcomeric protein titin (TTN) and its N-terminal fragment (N-TTN). Urinary N-TTN fragment level, obtained by proteolysis after muscle injury, reflects skeletal muscle atrophy or damage in different pathologies (Oshida et al., 2019; Miyoshi et al., 2020; Nakanishi et al., 2020) and has been proposed to monitor muscles deterioration in Duchenne muscular dystrophy (Awano et al., 2018) or the exercise-induced muscle damage (Kanda et al., 2017), although its value is currently being debated and needs more validation studies (Tanihata and Minamisawa, 2023).

The serum concentration of N-TTN has also been suggested as a marker of muscle disfunction, although there is limited data on this biofluid (Sun et al., 2014; Vassiliadis et al., 2012; Zybek-Kocik et al., 2021).

Other attractive potential biomarkers for sarcopenia are circulating microRNAs. By binding to the 3′untranslated region (3′UTR) of mRNAs, they are involved in a variety of biological processes through post transcriptional regulation of gene expression (Lee and Ambros, 2001; Lu and Rothenberg, 2018). MiRNAs expression is tissue and cell-type specific (Kim et al., 2014), but they can be detectable also in biofluids, such as blood, where they are involved in intercellular communication (Valadi et al., 2007) and may reflect physiological or pathological processes that occur in the tissue (Siracusa et al., 2018).

Several lines of evidence have shown their involvement in muscle development, repair and regeneration (Brzeszczynska et al., 2020), as well as in muscle functions and adaptations to physical exercise as to rehabilitation treatment (Davidsen et al., 2011); in addition, many studies have suggested microRNAs as potential biomarkers for sarcopenia, although further research is needed to validate and better verify the possible underlying mechanisms (Yanai et al., 2020).

Therefore, the primary aim of this study is to investigate if TTN and N-TTN can be possible biomarkers of sarcopenia even when measured in serum, and to verify whether a structured rehabilitation program can modulate their expression. A better understanding of the value of circulating TTN and N-TTN, which reflect the systemic responses to stress and injury, may have important clinical implications for muscle-related diseases.

A secondary objective of this study is to analyze possible associations between titin and the circulating expression of miR-451a, a possible regulator of this protein.

Elucidating the significance of these circulatory biomarkers may play a crucial role in several areas, such as assessing of muscle health, predicting the prognosis of related diseases, and evaluating the outcomes of the recovery interventions.

## 2 Materials and methods

### 2.1 Patients and controls

One-hundred-sixty subjects were enrolled in the study: 70 sarcopenic patients (57 females and 13 males) and 90 aged- and sex-matched controls (69 females and 21 males). Subjects were recruited by the Palazzolo Institute and by the IRCCS Santa Maria Nascente Center, Fondazione Don Carlo Gnocchi, both in Milan, Italy. Patients were diagnosed as being sarcopenic according to the European Working Group on Sarcopenia in Older People (EWGSOP) criteria (Cruz-Jentoft et al., 2019). Subjects with cognitive impairment, that were clinically instable, had a concomitant diagnosis of neoplastic or neurodegenerative diseases, or were unable to participate safely in the intervention program were excluded from the study; additionally, individuals undergoing steroid therapy were excluded, as glucocorticoids are known to influence gene expression (Clayton et al., 2018; De Iudicibus et al., 2018), and may directly or indirectly alter the expression of various miRNAs.

Sarcopenic patients were undergoing a 30-day structured rehabilitation treatment, as previously described (Agostini et al., 2021; Agostini et al., 2023). Briefly, this program included two daily-session (40′ in the morning and 30′ in the afternoon) and was adapted to each patient’s clinical status and tolerance. It comprised assisted mobilization, progressive resistance training, and standing proprioceptive and balance exercises. Gait training started with assisted walking and progressed to unassisted walking, as tolerated. Evaluation of Short Physical Performance Battery (SPPB) (Guralnik et al., 1994) and right and left handgrip strength was performed before and after the rehabilitative treatment for each patient; exercise load and intensity were adjusted based on clinical evaluation and functional performance tests. Given the unavailability of instrumental methods for quantifying muscle mass in our clinical setting, direct measurement of muscle mass was not performed. Therefore, the diagnosis of severe sarcopenia was based on the presence of both low muscle strength and low physical performance, in accordance with EWGSOP2 diagnostic criteria.

The study was performed in accordance with the Declaration of Helsinki. All subjects provided informed consent. The study was approved by the ethics committee of IRCCS Fondazione Don Carlo Gnocchi ONLUS (n#9_04/04/2018).

### 2.2 Titin protein measurement in serum

Titin (TTN) and N-fragment titin (N-TTN) were measured in serum of all the enrolled subjects by using commercial enzyme-linked immunosorbent assay (ELISA), according to the manufacturers’ instructions (DBA Italia, Segrate, Italy for TTN, Prodotti Gianni, Milano, Italy for N-TTN). Sera were diluted 1:50 for TTN, and 1:4 for N-TTN. TTN concentration was expressed as ng/mL (sensitivity: 0.16 ng/mL), whereas N-TTN as pmol/L (sensitivity: 18.03 pmol/L).

### 2.3 Serum miRNA isolation, cDNA reverse transcription and miRNA quantification

Peripheral blood was collected from all the patients before and after rehabilitative treatment. Serum was obtained by centrifugation (2,000 *g* × 10′ at room temperature) and stored at −80°C until miRNAs isolation; absence of hemolysis was assessed by visual inspection and by spectrophotometric measurement of hemoglobin absorbance at 414 nm (Shah et al., 2016). MiRNAs were semi-automatically isolated from 200 µL of serum by a column-based kit (MiRNeasy serum/plasma kit, Qiagen GmbH, Hilden, Germany) using Qiacube (Qiagen), according to manufacturer’s protocol. Equal miRNA concentration, measured by Qubit fluorometer and Qubit™ microRNA Assay Kit (Qiagen), was used for reverse transcription with miRCURY LNA RT kit (Qiagen), as specified by the manufacturer instruction.

The volume (3 µL) and dilution of cDNA (1:25) were kept consistent for all samples throughout the study, to avoid variations due to sample differences and handling. The quantification of miR-451a was performed by droplet digital PCR (QX200, Bio-Rad, Hercules, CA, United States) with LNA™-specific primer (Qiagen, code YP02119305), and ddPCR EvaGreen Supermix (Bio-Rad), as previously described [30, 31]. QuantaSoft software, version 17.4.0917 (Bio-Rad) and QX software, version 1.2 (Bio-Rad) were used to quantified copies of miRNAs per wells. The miRNA concentration was expressed as copies/ng of extracted RNA. Thresholds were determined manually for each experiment, according to the negative controls, which included a no template control.

### 2.4 Statistical analysis

Statistical analyses were performed using commercial software MedCalc Statistical Software package (Version 11.5.0.0; Ostend, Belgium). Normally distributed data were expressed as mean ± standard deviation, and comparisons among groups were analyzed by Student’s t-test, when appropriate. Not-normally distributed data were expressed as median and interquartile range (IQR: 25th and 75th percentile), and comparisons were analyzed by Mann-Whitney U test, as appropriate, and with Wilcoxon signed-rank test for paired data. Correlations were analyzed using Spearman’s correlation coefficient. Receiver operating characteristics (ROC) and area under curve (AUC) were used to evaluate the potential of TTN, N-TTN and miR-451a to predict the effectiveness of rehabilitation treatment. p-values corresponding to ≤0.05 were statistically significant.

## 3 Results

### 3.1 Clinical parameters

Demographic and clinical characteristics of the study population are summarized in Table 1. Gender and age were similar in the two groups.

**TABLE 1:** Demographic and clinical characteristics of the individuals enrolled in the study.

Parameters	Sarcopenic patients	Healthy controls
N	70	90
Gender (M:F)	13:57	21:69
Age	77.1 ± 6.5	78.5 ± 9.0
SPPB before rehabilitation	1 (0–1)	—
SPPB after rehabilitation	2 (1.0–4.5)	—
Right handgrip before rehabilitation	18.8 ± 7.5	—
Right handgrip after rehabilitation	19.9 ± 7.7	—
Left handgrip before rehabilitation	16.0 ± 7.0	—
Left handgrip after rehabilitation	16.0 ± 7.2	—

Age (years) and handgrip values (Kg) are reported as mean ± standard deviation, whereas SPPB, values are reported as median (interquartile range). F, female; M, male; SPPB, short physical performance battery.

As expected, SPPB was significantly increased after rehabilitative treatment (p < 0.0001). Right handgrip was increased after rehabilitation, although without reaching statistical significance, whereas left handgrip was only barely modified. Supplementary Figure S1 shows the distribution of right and left handgrip strength values stratified by gender.

### 3.2 Titin proteins concentration in serum

TTN and N-TTN serum concentration was measured and compared between sarcopenic patients and healthy subjects. In sarcopenic patients, serum concentration of these proteins was evaluated before and after rehabilitation treatment.

**FIGURE 1:**
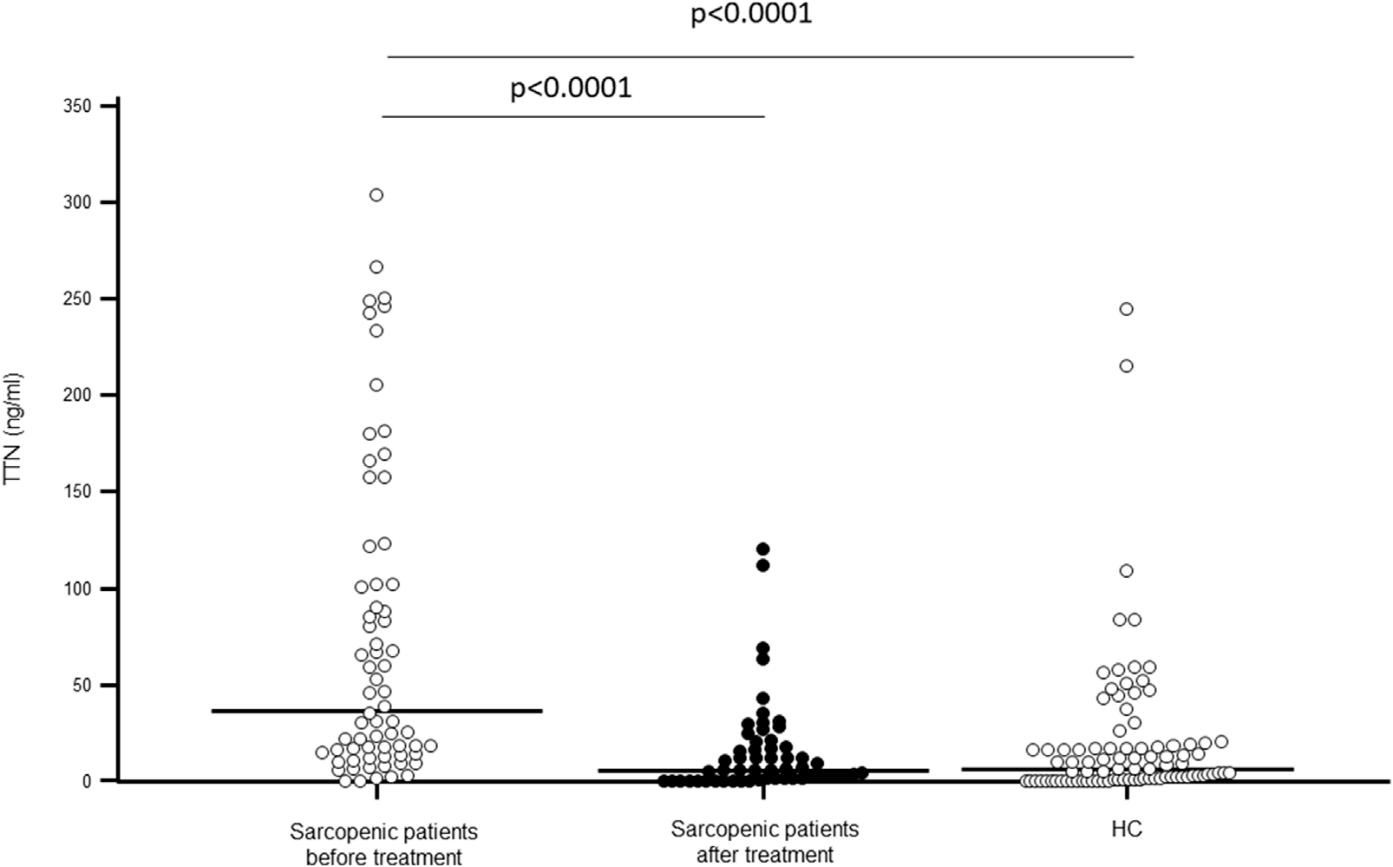
Titin (TTN) serum concentration (ng/mL) in sarcopenic patients before and after rehabilitative treatment, and in sex- and age-matched healthy controls (HC).

**FIGURE 2:**
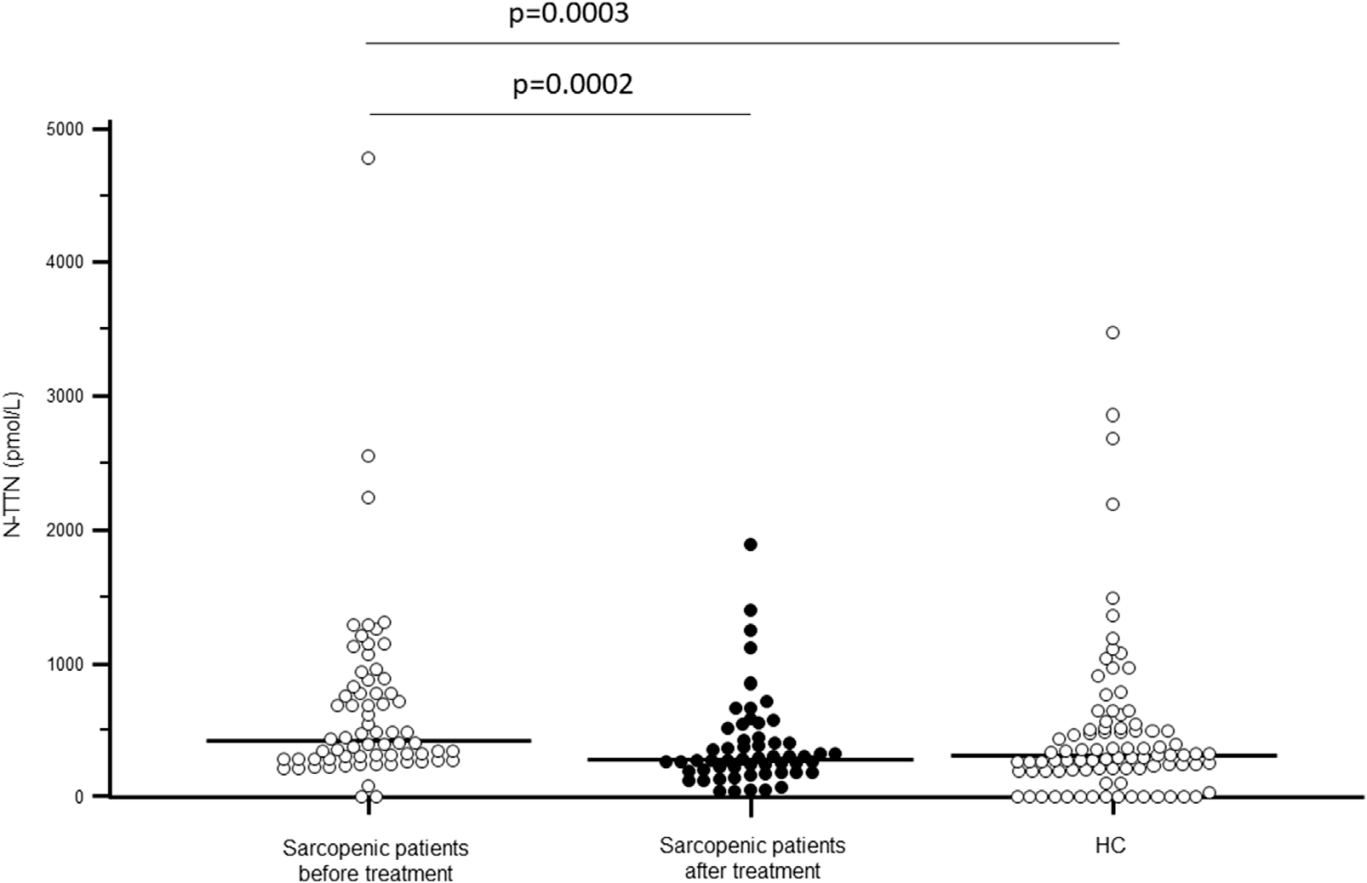
N-terminal fragment titin (N-TTN) serum concentration (pmol/L) in sarcopenic patients before and after rehabilitative treatment, and in sex- and age-matched healthy controls (HC).

As shown in Figure 1, TTN serum concentration was significantly higher in sarcopenic patients compared to HC (p < 0.0001); N-TTN serum concentration behaved in the same way, as significantly higher concentrations were detected in patients compared to HC (p = 0.003) (Figure 2).

Notably, TTN serum concentration was significantly decreased following rehabilitation (p < 0.0001), reaching the value of healthy controls. N-TTN serum concentration was significantly decrease as well following rehabilitation (p = 0.0002) and, similarly to what was observed for TTN, the statistical differences with the control group disappeared (Figures 1, 2).

A positive correlation between the change in N-TTN (delta-N-TTN) and the SPPB score after rehabilitation (Rho_s_ = 0.336; p = 0.024) was evidenced; moreover, a negative correlation was found between the baseline N-TTN value and the left handgrip strength value at the end of rehabilitative treatment (Rho_s_ = −0.297; p = 0.0362).

### 3.3 Prediction of miRNAs targeting titin 3′UTR and miR-451a selection

**FIGURE 3:**
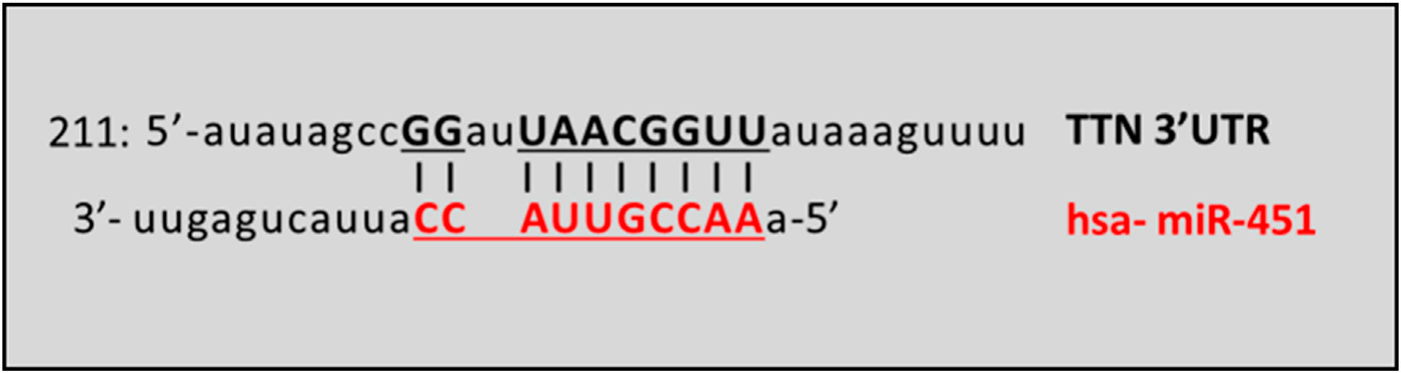
Schematic prediction of miR-451a binding site in the TTN transcript. The vertical lines denote the seed regions. TTN GeneBank Accession: NM_001256850.

MiRNAs targeting titin 3′UTR were predicted using three different computational algorithms: TargetScanHuman 8.0 (https://www.targetscan.org/vert_80/), miRdb Target mining (https://mirdb.org/mining.html) and microRNA Diana Lab (http://www.microrna.gr/). Among the miRNAs obtained and shared in all the three databases, we focused on miR-451a because it is involved in impairment of skeletal muscle functions, particularly in the context of aging, as sarcopenia and frailty (Mercken et al., 2013; Agostini et al., 2021; Agostini et al., 2023) or of genetic neuromuscular disorder as spinal muscular atrophy (Abiusi et al., 2021). The *in silico* binding prediction between miR-451a and 3′UTR of *TTN* mRNA is represented by Figure 3.

### 3.4 Circulatory miR-451a concentration

**FIGURE 4:**
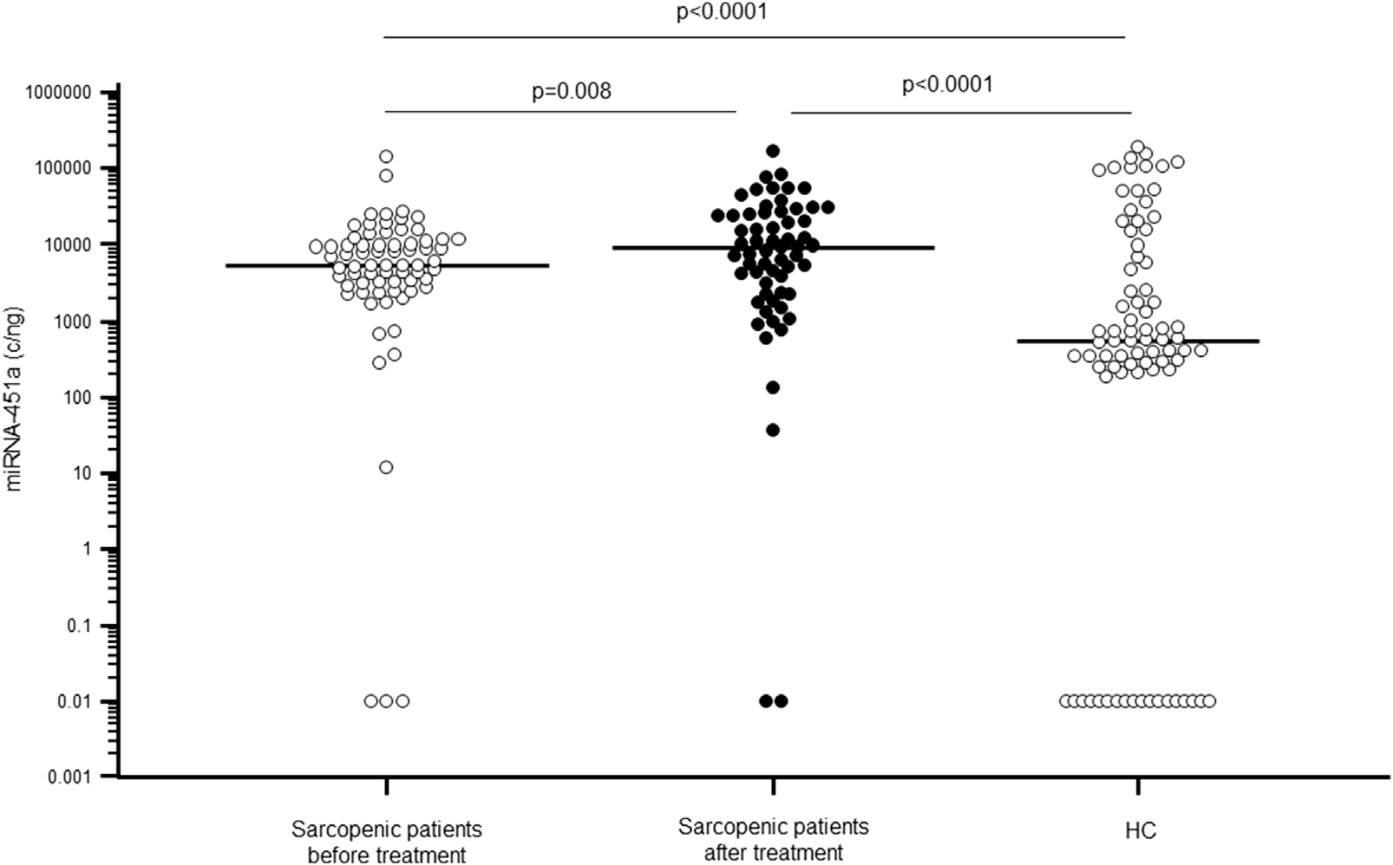
miR-451a serum concentration (copies/ng) in sarcopenic patients before (white dots) and after rehabilitative treatment (black dots), compared to sex- and age-matched healthy controls (HC).

Serum miR-451a concentration was significantly higher in sarcopenic patients (median: 5.46 × 10^3^; IQR: 2.92 × 10^3^–1.10 × 10^4^ c/ng) compared to HC (5.50 × 10^2^; 3.48 × 10^2^–8.08 × 10^3^ c/ng; p < 0.0001). Notably, the 30-day rehabilitative treatment was associated with a significant increase of miR-451a concentration (9.50 × 10^3^; 2.35 × 10^3^–2.44 × 10^4^ c/ng; p = 0.008) (Figure 4), confirming our previous results (Agostini et al., 2021).

Because statin treatment may be a potential confounder for miRNAs expressions (Lin et al., 2020), we analyzed miR-451a expression between patients with (37%) or without treatment, but no significant differences were observed for miR-451a.

Finally, an inverse correlation was observed between miR-451a and N-TTN expression both in sarcopenic patients (Rho = −0.337; p = 0.005) and HC subjects (Rho = −0.223; p = 0.046).

### 3.5 Predictive capability of circulatory TTN, N-TTN and miR-451a as biomarker of rehabilitative treatment outcome

**FIGURE 5:**
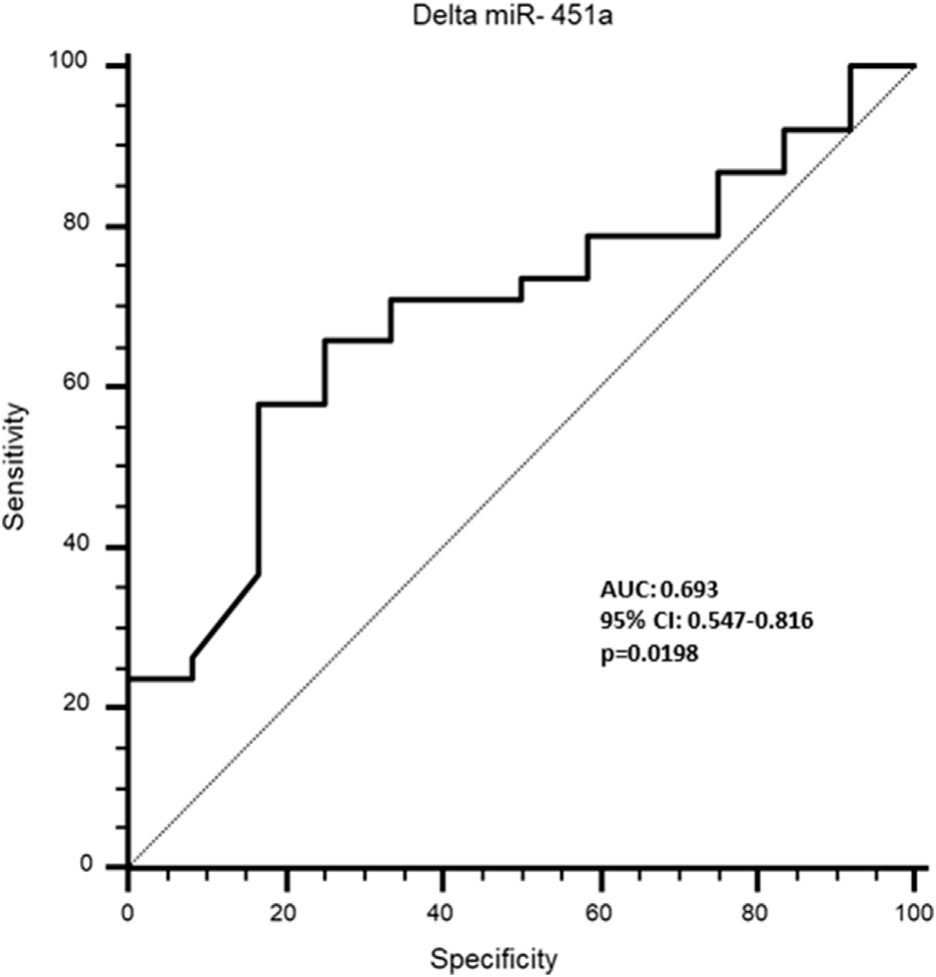
Receiver operating characteristics (ROC) curve to analyse the discriminatory value of delta miR-451a for rehabilitative effectiveness in sarcopenic patients.

Receiver Operating Characteristic (ROC) curve analysis was performed to evaluate the potential of TTN, N-TTN and miR-451a to predict the effectiveness of rehabilitation treatment, stratifying the sarcopenic patients on the base of changing in physical performance, measured by delta SPPB: the treatment was considered effective if delta SPPB≥1, and with no effect if delta SPPB = 0; as expected, no patient had the delta SPPB<0. This analysis did not show any significance for all the three parameters measured before the treatment. Moreover, when the delta expression before vs. after rehabilitative treatment was calculated, a significant discriminatory potential was observed for delta miR-451a (AUC: 0.693; specificity: 83.3; sensitivity: 57.9; p = 0.0198) (Figure 5), suggesting that it can be a valuable predictor of effectiveness of rehabilitation treatment; no significance was observed for delta TTN and delta N-TTN.

## 4 Discussion

This study analyzed expression of TTN and N-TTN proteins in serum as possible biomarkers for sarcopenia, and evaluated whether their expression levels change following a structured rehabilitation programme.

TTN is a large structural sarcomeric protein with a critical role in muscle elasticity, contraction, and structural integrity (Matsuo et al., 2019). N-TTN fragment, produced in damaged muscle by different proteolytic enzymes (as MMPs, Calpain-3), is released in blood and rapidly excreted in urine. The increase in the urinary N-TTN is considered an indirect measure of muscle injury and dysfunction of various origins, such as genetic mutation in neuromuscular disease DMD (Awano et al., 2018), malignancies or other diseases (Oshida et al., 2019; Miyoshi et al., 2020; Nakanishi et al., 2020; Zybek-Kocik et al., 2021; Varga et al., 2023), and also in case of mechanical damage during intense physical activity (Kanda et al., 2017); however, its effectiveness in early detection of muscle atrophy remains unclear, as a recent research demonstrated that urinary N-TTN is unable to identify early-stage muscle atrophy (Tanihata and Minamisawa, 2023).

Data regarding TTN and N-TTN in serum are limited (Vassiliadis et al., 2012; Zybek-Kocik et al., 2021); however, it has been suggested that their concentration in this biofluid may be related to muscle turnover induced by atrophy (Sun et al., 2014).

Our study showed, at our knowledge for the first time, that serum concentration of both TTN and its breakdown product, N-TTN, was significantly increased in sarcopenic patients compared to HC; these findings support the hypothesis that the serum measurement of these two proteins may be a useful marker for monitoring change in muscle mass and function associated with several clinical conditions, as, *i.e.*, inactivity associated with hospitalization or disease state (Nakanishi et al., 2020).

Importantly, we observed that serum concentration of these proteins decreased significantly in patients following rehabilitation. The effect of physical exercise on titin in muscle has been previously investigated, with evidence of exercise-induced fragmentation (Macaluso et al., 2014), and post-translational modifications such as phosphorylation (Muller et al., 2014) which can modulate the mechanical performance of muscle, participating to adaptation after chronic or acute exercise (Kruger and Kotter, 2016). It is plausible to infer that the downregulation of TTN and N-TTN concentrations observed in our study after rehabilitation reflects an associated improvement in muscle function; this reduction may also result from exercise-induced modulation of proteolytic pathways, including calpain and MMPs activity, as previously reported (Hyatt and Powers, 2020; de Sousa Neto et al., 2018). This interpretation is supported by the overall increased in physical performance as indicated by SPPB score measured in sarcopenic patients after treatment. Analyzing the correlation between N-TTN and functional outcomes, although no direct association was obtained between TTN or N-TTN and SPPB, we observed that the increasing N-TTN fragments (delta N-TTN) during rehabilitation was associated with higher SPPB scores at discharge, suggesting that treatment may induce TTN fragmentation and muscle protein turnover, that may result in increased functionality. Moreover, our analysis showed also that individuals with higher initial N-TTN levels tended to have lower handgrip strength at the end of rehabilitation treatment. These observations support previous hypotheses regarding the role of TTN and its fragmentation in adaptive muscle remodeling (Kruger and Kotter, 2016). However, these correlations need a validation in larger and longitudinal studies, including other muscle mass synthesis/degradation and mass biomarkers (e.g., creatine kinase, Appendicular Lean Mass index).

It is important to note that these two biomarkers might be related to other indices of muscle strength and power: future studies based on data obtained by, *i.e.*, magnetic resonance imaging or bioelectrical impedance analysis or by more invasive procedure like muscle biopsies, could better clarify the relation between TTN, N-TTN and functional muscle improvement after rehabilitation.

The complex mechanisms responsible for the dynamic regulation of TTN expression in response to physical exercise are only beginning to be understood; in particular it is known that exercise can lead to multiple tissue-specific epigenetic modifications that regulate gene expression (reviewed in Jacques et al., 2019), including DNA methylation, histone modifications, along with non-coding RNAs as miRNAs. Because miRNAs can be released in circulation from tissues, they are a promising class of biomarkers for detecting muscle damage, assessing exercise adaptation and also evaluating the effectiveness of rehabilitation intervention across different patient population (Fochi et al., 2020).

Therefore, using computational algorithms we have found that miR-451a can target 3′UTR of *TTN* gene. MiR-451a is a highly conserved miRNA, located on chromosome 17q11.2, and it is involved in the regulation of various cellular functions; this miRNA is abundant in erythrocytes (Azzouzi et al., 2015), where it is crucial for red blood cells homeostasis (Rasmussen et al., 2010).

In addition to being deregulated in different types of tumors (Pan et al., 2013; Gupta et al., 2025; Moni et al., 2025), in neurodegenerative diseases like Alzheimer’s (Duan et al., 2024), coronary artery disease (Andiappan et al., 2024) and IgA nephropathy (Zhang et al., 2024), miR-451a has been reported to be involved in muscle biology through a variety of mechanisms: one of its target is the SPARC (Secreted Protein Acidic Rich in Cysteine) protein (Munk et al., 2019), an exercise induced molecule (Ghanemi et al., 2022) with crucial role in muscle development and regeneration (Jorgensen et al., 2009; Petersson et al., 2013); its expression in skeletal muscle increases with age in association with the decline of muscle regeneration (Mercken et al., 2013); it is upregulated in circulation for effect of acute (Nair et al., 2020) or long-term exercise in healthy subjects (Li et al., 2020). Besides SPARC, other possible targets involved in muscle biology are peroxisome proliferator-activated receptor gamma coactivator 1α (PGC-1α), which regulates mitochondrial biogenesis and oxidative capacity (Gaal et al., 2022), AMP-activated protein kinase (AMPK), a key regulator of energy metabolism in skeletal muscle (Massart et al., 2016), and bone morphogenetic protein 6 (BMP6), involved in muscle growth, maintenance, and regeneration (Lu et al., 2019).

Finally, miR-451a expression increases in muscle biopsies of healthy subjects in association with low response after resistance exercise training, namely, without muscle mass gain, supporting the hypothesis that this miRNA is involved in modulation of genes related to muscle adaptation and remodeling after strength training (Davidsen et al., 2011).

Our results confirmed that miR-451a serum concentration is significantly increased in sarcopenic patients compared to healthy subjects and that rehabilitation stimulates its production, consistently with results obtained in previous works (Agostini et al., 2021; Agostini et al., 2023). To note, a negative correlation between miR-451a and N-TTN was detected in both HC and sarcopenic patients, supporting a miR-451a regulatory activity on this protein; further studies are needed to confirm and clarify this relation.

Based on our results, it is possible to hypothesize that the increased age-related expression of miR-451a in muscle cells contributes to degeneration of muscle mass in sarcopenic patients with release miR-451a in blood. Moreover, according with previous researches (Li et al., 2020; Nair et al., 2020) our findings confirm that physical exercise modulates the circulatory expression of miR-451a.

An interesting result obtained by ROC analysis is that the variation of miR-451a expression in serum of sarcopenic patients before vs. after treatment is related to changing of physical performance (delta SPPB), suggesting that mir-451a measurement may predict the efficacy of the rehabilitation.

It is important to note that muscle biology and function are regulated by complex and multifactorial mechanisms. Therefore, future studies are needed to integrate our findings on miR-451a and TTN with additional muscle-specific miRNAs and biomarkers, aiming to obtain a comprehensive molecular profile with strong diagnostic and prognostic value for sarcopenia. Moreover, to increase the diagnostic specificity of miR-451a and minimize the risk of confounding interpretations, it would be valuable to include systemic biomarkers such as circulating tumor markers and neurodegeneration-related markers in future analyses. This combined approach could help discriminate sarcopenia-related changes from those associated with other pathological conditions. The present study presents some limitations: 1) the interaction between miR-451a and TTN is based only by *in silico* prediction: further *in vitro* studies are necessary to verify this interaction and the biological effects, and to understand the mechanisms underlying the effect of rehabilitation in sarcopenic patients; 2) as molecules concentration in serum can be physiologically transient and not stable, further longitudinally studies in which the miR-451a, TTN and N-TTN levels are monitored day by day are necessary to confirm our results; 3) the study cohort is limited: further studies on bigger cohorts will be useful to verify if the combined measurement of different markers involved in muscle biology could increase the predictive capability of rehabilitative treatment outcome; 4) in accordance with EWGSOP2 guidelines (Cruz-Jentoft et al., 2019), in our study sarcopenia was identified based on low muscle strength (handgrip strength) and low physical performance (SPPB). Although direct assessment of muscle mass (using dual-energy X-ray absorptiometry or bioelectrical impedance analysis) is recommended, such equipment was not available in our geriatric setting; therefore, muscle mass was not measured. Nonetheless, our diagnostic approach remains consistent with EWGSOP2, which allows for the identification of severe sarcopenia based on functional criteria alone when direct muscle mass assessment is not feasible.

In conclusion, our results suggest that serum TTN, N-TTN and miR-451a could be valid biomarkers for sarcopenia and support the hypothesis that modulation of non-coding RNAs is a pivotal epigenetic mechanism involved in recovery after rehabilitative treatment of sarcopenic patients.

## Data Availability

The raw data supporting the conclusions of this article will be made available by the authors, without undue reservation.
