# Supplementary material for: Circulatory titin and miR-451a are possible sarcopenia biomarkers in elderly people
Source: Front Aging. 2025 Jun 12;6:1587438. doi: 10.3389/fragi.2025.1587438 (PMC12198232; doi:10.3389/fragi.2025.1587438)
Supplement: Supplementary file 1 [file DataSheet1.pdf]

Circulatory titin and miR-451a are possible sarcopenia biomarkers in elderly people

Roberta Mancuso[1#], Lorenzo Agostino Citterio[1#a], Simone Agostini[1*], Rossella Miglioli[1], Riccardo Nuzzi[1], Laura Antolini[2], Fabio Trecate[1], Mario Clerici[1,3]

[1]IRCCS Fondazione Don Carlo Gnocchi ONLUS, Milan, Italy.

[2]Department of Health Sciences, University of Milano-Bicocca, Monza, Italy.

[3]Department of Pathophysiology and Transplantation, University of Milan, Italy

[a]Present address: University of Pavia, PhD National Programme in One Health approaches to infectious diseases and life science research, Department of Public Health, Experimental and Forensic Medicine, Pavia, Italy, and University of Milan, Department of Biomedical and Clinical Sciences, Milan, Italy

*** Correspondence:**
Simone Agostini
sagostini@dongnocchi.it

#equal contribution and first authorship

Supplementary Material

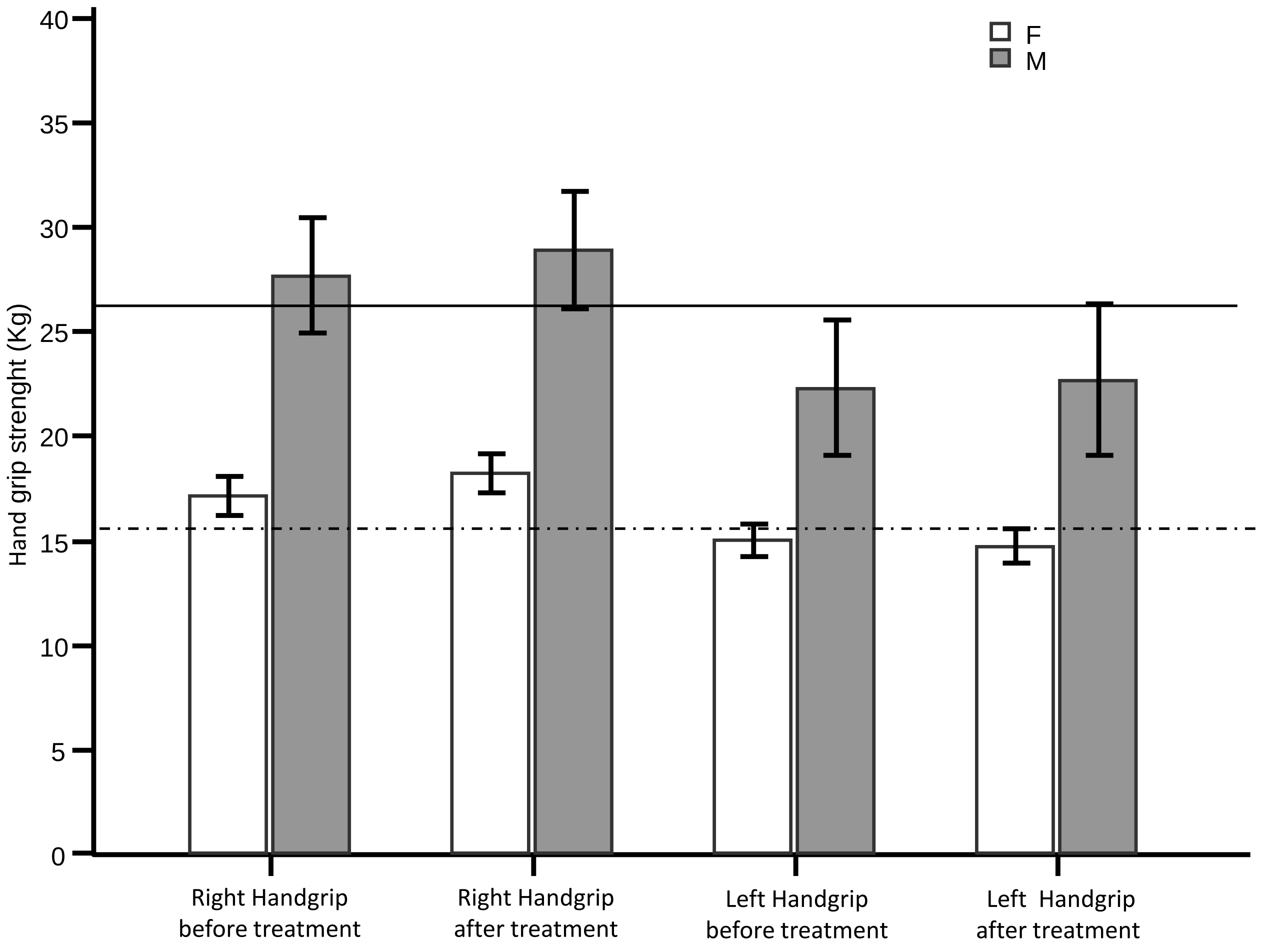

Supplementary Figure 1. Distribution of right and left handgrip strength values stratified by gender. Bars represent mean values and standard errors. Diagnostic cut-off values for males (solid line) and females (dotted line) are indicated, based on EWGSOP2 criteria.
